# mTOR Knockdown in the Infralimbic Cortex Evokes A Depressive-like State in Mouse

**DOI:** 10.3390/ijms22168671

**Published:** 2021-08-12

**Authors:** Emilio Garro-Martínez, Maria Neus Fullana, Eva Florensa-Zanuy, Julia Senserrich, Verónica Paz, Esther Ruiz-Bronchal, Albert Adell, Elena Castro, Álvaro Díaz, Ángel Pazos, Analía Bortolozzi, Fuencisla Pilar-Cuéllar

**Affiliations:** 1 Centro de Investigación Biomédica en Red de Salud Mental (CIBERSAM), Instituto de Salud Carlos III, 28029 Madrid, Spain; egamarrone@gmail.com (E.G.-M.); mneusfl@gmail.com (M.N.F.); florensae@unican.es (E.F.-Z.); senserrichj@unican.es (J.S.); veronica.paz@iibb.csic.es (V.P.); esther.ruiz@iibb.csic.es (E.R.-B.); adella@unican.es (A.A.); castroe@unican.es (E.C.); diazma@unican.es (Á.D.); pazosa@unican.es (Á.P.); analia.bortolozzi@iibb.csic.es (A.B.); 2 Instituto de Biomedicina y Biotecnología de Cantabria (IBBTEC), Universidad de Cantabria-CSIC-SODERCAN, 39011 Santander, Spain; 3 Departamento de Fisiología y Farmacología, Universidad de Cantabria, 39011 Santander, Spain; 4 Department of Neuroscience and Experimental Therapeutics, Instituto de Investigaciones Biomédicas de Barcelona (IIBB-CSIC), 08036 Barcelona, Spain; 5 Systems Neuropharmacology Group, Instituto de Investigaciones Biomédicas August Pi i Sunyer (IDIBAPS), 08036 Barcelona, Spain

**Keywords:** mTOR, infralimbic cortex, behavioral despair, BDNF, neurotransmitter

## Abstract

Fast and sustained antidepressant effects of ketamine identified the mammalian target of rapamycin (mTOR) signaling pathway as the main modulator of its antidepressive effects. Thus, mTOR signaling has become integral for the preclinical evaluation of novel compounds to treat depression. However, causality between mTOR and depression has yet to be determined. To address this, we knocked down mTOR expression in mice using an acute intracerebral infusion of small interfering RNAs (siRNA) in the infralimbic (IL) or prelimbic (PrL) cortices of the medial prefrontal cortex (mPFC), and evaluated depressive- and anxious-like behaviors. mTOR knockdown in IL, but not PrL, cortex produced a robust depressive-like phenotype in mice, as assessed in the forced swimming test (FST) and the tail suspension test (TST). This phenotype was associated with significant reductions of mTOR mRNA and protein levels 48 h post-infusion. In parallel, decreased brain-derived neurotrophic factor (BDNF) expression was found bilaterally in both IL and PrL cortices along with a dysregulation of serotonin (5-HT) and glutamate (Glu) release in the dorsal raphe nucleus (DRN). Overall, our results demonstrate causality between mTOR expression in the IL cortex and depressive-like behaviors, but not in anxiety.

## 1. Introduction

Major depressive disorder (MDD) is a chronic, recurrent, and multifactorial psychiatric disorder that places the subject’s life at risk and has become the leading cause of disability worldwide in terms of disease [1]. MDD is characterized by a series of physiological, psychological, and behavioral symptoms such as depressed mood, sleep or appetite disturbances, loss of interest and pleasure, or the impairment of executive functions, among others.

As the neurobiological basis of this pathology is still unknown, several hypotheses have been proposed to date. One of the most relevant is the neurotrophic/neuroplastic hypothesis of depression, which postulates that a reduction in neurotrophic factors (such as the brain-derived neurotrophic factor, BDNF), in brain areas such as the hippocampus and the prefrontal cortex, underlies the neuronal atrophy associated with this disease in preclinical [2,3,4] and clinical studies [5,6]. In addition, increased hippocampal BDNF levels were associated with antidepressant response in human studies [5], as well as in preclinical studies after chronic treatment with classic antidepressant drugs [7,8,9,10,11,12,13], or after acute treatment using fast-acting antidepressants such as ketamine [14,15,16,17,18].

The fast antidepressant effect of ketamine, an *N*-methyl-d-aspartate (NMDA) receptor antagonist, has been attributed to the activation of the mammalian target of rapamycin (mTOR) signaling pathway in rat [14,19]. mTOR is a serine/threonine-protein kinase that was first described in yeast as the pharmacological target of rapamycin [20]. In mammals, there are two different complexes, mTORC1 and mTORC2 [21], which control various aspects of cell physiology. In the central nervous system, the activation of mTORC1 triggers the phosphorylation of several effectors such as the ribosomal protein S6 kinase 1 (p70S6K), or the eukaryotic translation initiation factor 4E-binding protein (4E-BP1) [22], inducing protein translation. These proteins are located in the neuronal soma and dendrites, specifically in the synapses, where they colocalize with the postsynaptic density protein-95 (PSD-95), suggesting a postsynaptic location of the pathway [23]. Following activation of the mTOR pathway, there is an increased synthesis of proteins such as PSD-95, the GluA1 subunit of the AMPA receptor, and presynaptic proteins such as synapsin 1 or the activity-regulated cytoskeleton-associated protein (Arc) [14,24,25]. These proteins participate in synaptic plasticity processes such as the formation, maturation, and function of new dendritic spines [14], memory processes [22], or long-term potentiation (LTP) [23,26], which are impaired in MDD.

Mental disorders are defined as “connectopathies” with a complex pathological mechanism at the level of circuits and their communication [27]. Neuroimaging studies in MDD patients show functional and structural connectivity disruptions in the cingulate cortex, the hippocampus, and the prefrontal cortex, among others [28,29]. The ventral anterior cingulate cortex (vACC, Brodman area 25, Cg25) has a special interest since clinical studies reported functional hyperactivity of this area in MDD patients [28,29,30]. Likewise, a moderate small interfering RNAs (siRNA)-induced reduction of astrocytic GLAST and GLT-1 expression in the mouse infralimbic cortex (IL, rodent counterpart to human vACC), markedly increased local glutamatergic neurotransmission and evoked a depressive-like phenotype [31,32]. Moreover, different preclinical studies support the importance of the IL cortex in the antidepressant-like effects induced by deep brain stimulation (DBS) [33,34,35,36], (2R,6R)-hydroxynorketamine [37], ketamine, or optogenetic stimulation [38]. The activation of the IL cortex reverses changes in the dorsal raphe nucleus (DRN) of animals subjected to the chronic social defeat depression model [34], reinforcing the critical role of this brain area and its outputs to the midbrain in the regulation of emotion and stress responses.

Previous studies have reported the downregulation of the mTOR signaling pathway in postmortem human brain samples from subjects with MDD [39], as well as in PFC, hippocampus, and amygdala of several animal models of depression including the olfactory bulbectomy, chronic unpredictable stress (CUS), and chronic corticosterone exposure [40,41,42,43,44]. In order to examine the role of the mTOR pathway in the IL cortex as a neurobiological substrate in MDD, we evaluated in this study the effects of the mTOR knockdown in mouse IL cortex on the anxious- and depressive-like behavior, the expression of synaptic plasticity markers as the brain-derived neurotrophic factor (BDNF) using in situ hybridization, and used in vivo microdialysis to explore the evoked adaptive changes in cortico–subcortical circuits leading to the emergence of a depressive-like phenotype.

## 2. Results

### 2.1. Acute Unilateral mTOR Silencing in the Infralimbic Cortex Induces a Depressive- but Not Anxiety-like Behavior

**Figure 1:**
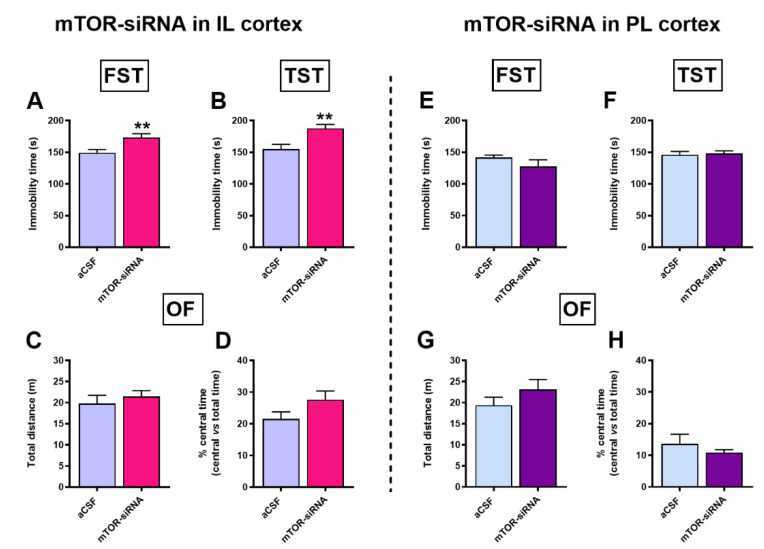
Acute mTOR knockdown in the infralimbic, but not in the prelimbic cortex, induces a depressive-, but not anxious-like behavior. Depressive-like responses were examined using (**A**) forced swimming test and (**B**) tail suspension test, respectively, 24 h and 48 h after unilateral mTOR-siRNA infusion in the IL cortex. No changes were observed in (**C**) locomotion or (**D**) anxiety-related behavior in intra-IL mTOR knockdown mice evaluated in the open-field test 24 h after siRNA infusion. (**E**–**H**) No behavioral changes were observed when mTOR was knocked down in the PrL cortex. Results are presented as mean ± S.E.M. Unpaired Student’s *t*-test, ** *p* < 0.01. n = 7–9 animals per group. aCSF: artificial cerebrospinal fluid; IL: infralimbic; PrL: prelimbic; FST: forced swimming test; TST: tail suspension test; OF: open-field test; siRNA: small interfering RNA.

We examined the role of mTOR knockdown in mPFC on depressive- and anxiety-like behavior by a single unilateral intracerebral infusion selectively into the IL or PrL cortices. mTOR-siRNA infusion (40 µg/μL siRNA pool) into the IL cortex evoked a depressive-like behavior measured in the FST and TST, 24 and 48 h post-infusion, respectively (Figure 1A,B). The immobility times were significantly higher compared to the control groups in the FST (mTOR-siRNAs: 173.0 ± 6.2 s vs. aCSF: 149.2 ± 5.2 s, *p* < 0.01), and in the TST (mTOR-siRNAs: 187.7 ± 6.3 s vs. aCSF: 155.2 ± 7.4 s, *p* < 0.01). The observed increase in immobility time was not due to an overall reduction in locomotor activity, as the total distance traveled in the open field was similar between both groups (Figure 1C). In addition, we evaluated the total time spent within the central area of the arena to preliminary study putative anxiety-related behaviors using the open-field test paradigm. No differences were observed between the groups (Figure 1D).

The infusion of mTOR-siRNAs in the PrL cortex (Figure 1E–H) did not produce changes in any of the tests used to evaluate depressive- or anxiety-like behaviors. Likewise, a comparable behavioral profile was observed after intra-IL NS-siRNA infusion compared to control mice receiving intra-IL aCSF (Figure S1).

Thus, the unilateral acute infusion of mTOR-siRNA in the IL cortex induces a depressive-like behavior, as evidenced by the increased behavioral despair.

### 2.2. Intra-Infralimbic mTOR-siRNA Infusion Reduces mTOR mRNA Expression and Protein Levels

**Figure 2:**
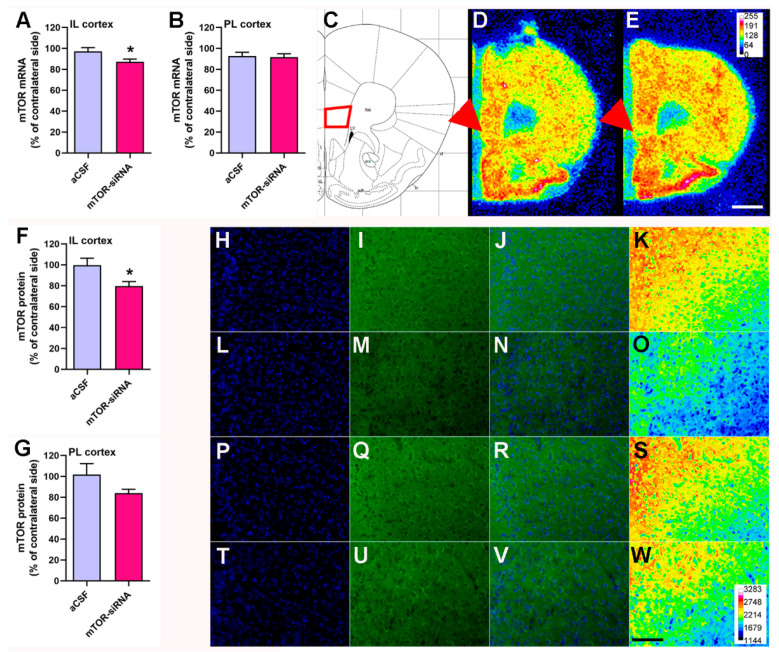
mTOR downregulation after intracerebral administration of siRNAs targeting mTOR in the infralimbic cortex (**C**). mTOR levels in (**A**,**F**), the IL, and (**B**,**G**) the PrL cortex were evaluated by (**A**–**E**) in situ hybridization, and (**F**–**W**) mTOR protein expression by immunofluorescence, after unilateral infusion into the IL cortex. Data are expressed as mean ± S.E.M. Unpaired Student’s *t*-test, * *p* < 0.05. *n* = 4–5 animals per group. Representative coronal brain sections containing IL cortex showing mTOR mRNA expression in (**D**) aCSF-infused and (**E**) mTOR-siRNA-infused mice as assessed by in situ hybridization. Representative immunofluorescent images of the mTOR protein levels in (**H**–**K**) the IL and (**P**–**S**) the PrL cortex of vehicle-infused mice, and in (**L**–**O**) the IL and (**T**–**W**) the PrL cortex of mTOR-siRNA-infused mice. Images show (**H**,**L**,**P**,**T**) DAPI, (**I**,**M**,**Q**,**U**) mTOR, (**J**,**N**,**R**,**V**) merged, and (**K**,**O**,**S**,**W**) heat-map image. Scale bar in E: 1 mm. Scale bar in W: 100 µm. Heat-map calibration bar in E and W showing the intensity values. aCSF: artificial cerebrospinal fluid; IL: infralimbic; PrL: prelimbic; siRNA: small interfering RNA.

The mTOR downregulation was confirmed using in situ hybridization (Figure 2A–E) and immunohistochemistry (Figure 2F–W) procedures. mTOR-siRNA infusion in the IL cortex significantly reduced mTOR mRNA expression in the IL (two-tailed unpaired *t*-test, t8 = 2.315; *p* < 0.05) (Figure 2A,E), but not in the PrL cortex (Figure 2B,E).

In parallel, we found that intra-IL mTOR-siRNA infusion induced a significant reduction of mTOR protein levels locally in the IL cortex (Figure 2F,L–O) compared to control mice (Figure 2H–K) (two-tailed unpaired *t*-test, t8 = 2.524; *p* < 0.05), without affecting mTOR expression in the PrL cortex (Figure 2G,P–W).

The unilateral infusion of mTOR-siRNA in the IL cortex induces a reduction in mTOR mRNA and protein levels in the ipsilateral side 48 h after siRNA infusion.

### 2.3. Reduced BDNF mRNA Expression in Medial Prefrontal Cortex after mTOR Knockdown in Infralimbic Cortex

**Figure 3:**
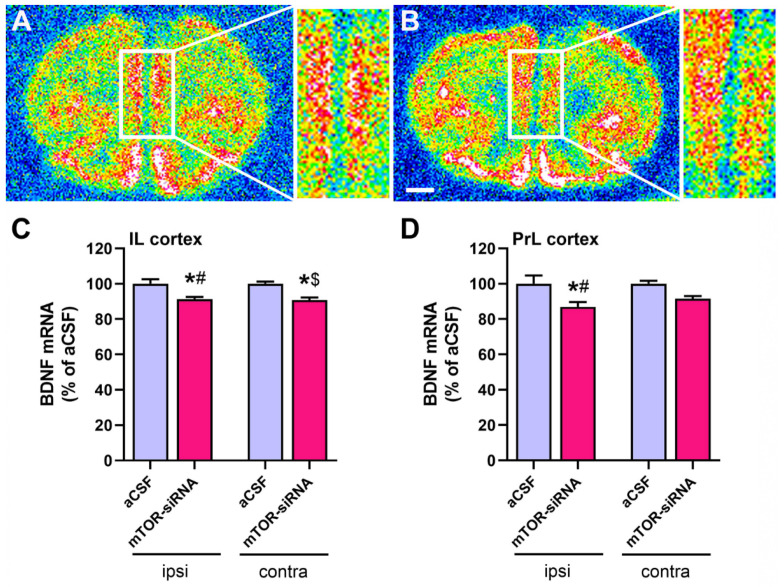
mTOR knockdown in infralimbic cortex reduces BDNF transcription in medial prefrontal cortex. Representative coronal brain sections showing BDNF mRNA expression in (**A**) aCSF-treated and (**B**) mTOR-siRNA-treated mice, as assessed by in situ hybridization. Effect of local mTOR-siRNAs infusion in the IL cortex on BDNF mRNA expression in the mPFC: (**C**) IL and (**D**) PrL areas. Data are expressed as mean ± S.E.M. Two-way ANOVA followed by a Bonferroni post hoc test, * *p* < 0.05 compared to their respective aCSF group; # *p* < 0.05 compared to the contralateral aCSF group; $ *p* < 0.05 compared to the ipsilateral aCSF group. *n* = 5 animals per group. Scale bar: 1 mm. aCSF: artificial cerebrospinal fluid; BDNF: brain-derived neurotrophic factor; contra: contralateral to the infusion site; ipsi: ipsilateral to the infusion site; IL: infralimbic; PrL: prelimbic; siRNA: small interfering RNA.

Given that brain BDNF levels are critical in the neurobiology of MDD, as well as in the antidepressant effects [45,46], we also examined the BDNF expression in the intra-IL mTOR knockdown mice. Moreover, as the mTOR-siRNA was infused in the IL cortex unilaterally, we also considered the brain side as a variable. mTOR knockdown in the IL cortex produced a significant reduction of BDNF mRNA expression in both brain sides (ipsi- and contralateral IL cortices) (Figure 3B,C), and in the ipsilateral PrL cortex (Figure 3B,D) 48 h post-infusion. Two-way ANOVA analyzing the effect of the treatment and the brain side factors revealed a significant effect of treatment on the BDNF expression in the IL cortex (F(1,16) = 26.41, *p* < 0.001) (Figure 3C), and in the PrL cortex (F(1,16) = 13.65, *p* < 0.01) (Figure 3D).

The unilateral infusion of mTOR-siRNA in the IL cortex induces a bilateral reduction in BDNF mRNA levels in the IL cortex, and in the ipsilateral side in the PrL cortex.

### 2.4. Extracellular Serotonin and Glutamate Levels in Dorsal Raphe Nucleus after mTOR Knockdown in Infralimbic Cortex

**Figure 4:**
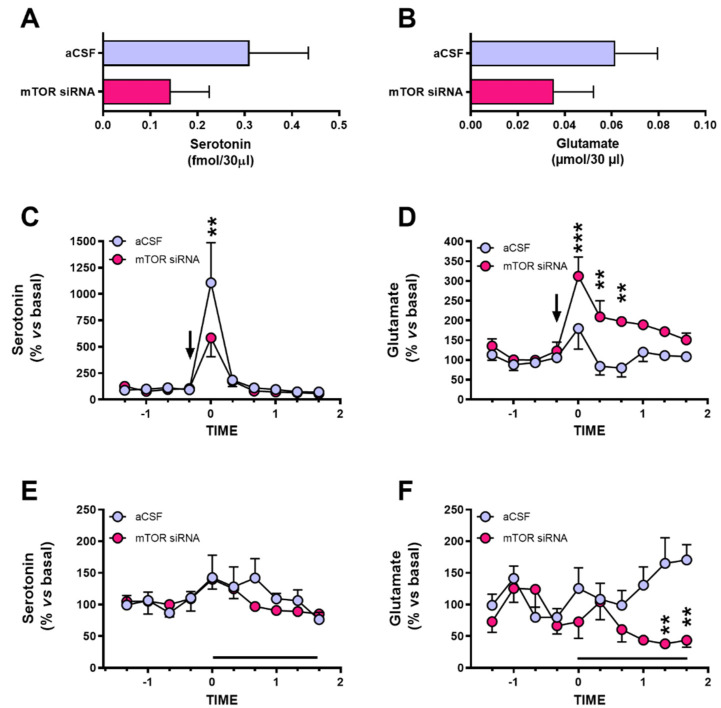
Dysregulation of extracellular serotonin and glutamate neurotransmitters in the dorsal raphe nucleus after acute infralimbic-mTOR knockdown in mice. Baseline extracellular values of (**A**) serotonin and (**B**) glutamate in DRN. The effects of local 50 μM veratridine on extracellular (**C**) serotonin and (**D**) glutamate levels, and intra-DRN 100 μM NBQX infusion on extracellular (**F**) 5-HT and (**E**) glutamate levels in control and mTOR-siRNAs mice. Data are represented as mean ± SEM. Two-way ANOVA followed by Bonferroni post hoc test (** *p* < 0.01, *** *p* < 0.001). *n* = 4–5 animals per experimental group. 5-HT: serotonin; aCSF: artificial cerebrospinal fluid; NBQX: 2,3-dihydroxy-6-nitro-7-sulfamoyl-benzo(f)quinoxaline; siRNA: small interfering RNA.

Glutamatergic projections from the IL cortex regulate the 5-HTergic neuronal activity in the DRN, and alterations of the IL-DRN circuitry function are implicated in depressive-like behaviors [31,38,47]. Therefore, extracellular 5-HT and glutamate levels were assessed in the DRN of mice after acute mTOR knockdown in the IL cortex using in vivo microdialysis procedures. mTOR knockdown in the IL cortex did not alter the extracellular basal levels of 5-HT and glutamate in the DRN compared to control animals (Figure 4A,B). The local administration of the depolarizing agent veratridine (50 μM) in the DRN revealed opposite effects on presynaptic releasable pools of 5-HT and glutamate since extracellular levels of 5-HT were lower (Figure 4C), while those of glutamate were higher (Figure 4D) in the IL cortex of mTOR knockdown animals compared to control mice. Two-way ANOVA showed a significant effect of time (F(9,72) = 12.71, *p* < 0.001) for 5-HT, whereas significant effects of mTOR siRNAs (F(1,8) = 18.57, *p* < 0.01), time (F(9,72) = 8.00, *p* < 0.001), and interaction of both factors (F(9,72) = 2.62, *p* < 0.05) were found for glutamate.

The local application of the AMPA receptor antagonist NBQX (100 μM) in the DRN of IL-mTOR silenced mice did not modify the extracellular 5-HT levels in DRN (Figure 4E) while it reduced the DRN glutamate levels (Figure 4F). Two-way ANOVA revealed a significant effect of time (F(9,72) = 2.33, *p* < 0.05) for 5-HT, and a significant effect of mTOR-siRNAs (F(1,8) = 10.66, *p* < 0.05) and the interaction of both factors (F(9,72) = 3.250, *p* < 0.01) for glutamate.

The unilateral infusion of mTOR-siRNA in the IL cortex induces the dysregulation in the serotonergic and glutamatergic neurotransmission in the DRN.

## 3. Discussion

Herein, we demonstrated that acute mTOR knockdown in the IL cortex, induced by the intracerebral administration of mTOR-siRNA, elicited a depressive-like state in mice, which was maintained for at least 48 h without affecting anxiety-like behavior. The acute mTOR downregulation in the IL cortex leads to reduced BDNF mRNA levels in mPFC, together with impaired 5-HT and glutamate neurotransmission in the DRN, disturbances linked to neuropathophysiology of depression, as reported in preclinical [31,36,48,49] and clinical [50] studies. Our findings highlight the importance of mTOR signaling in the IL cortex for adequate mPFC-DRN circuit functioning.

In the present mouse model, a single unilateral mTOR-siRNA dose in the IL cortex was sufficient to reduce mTOR expression (mRNA and protein), ultimately inducing a depressive-phenotype, and this is in line with the lower mTOR protein levels found in the mPFC of patients with MDD [39]. In addition, the overexpression of the mTOR negative regulator REDD1 (regulated in development and DNA damage responses-1) in mPFC was associated with depressive-like behavior in rats [41]. Moreover, the long-term oral administration of mTOR inhibitors (i.e., rapamycin) in rodents also induced a depressive-like state characterized by behavioral despair [51,52] and anhedonia [51]. In line with these observations, pilot studies in our lab using subchronic mTOR siRNA infusion showed both behavioral despair and anhedonia in mice (data not shown). However, neither the subchronic [51] nor the acute [51,53,54] systemic administration of rapamycin in rat induced depressive-like effects. It is worth mentioning that some authors reported an antidepressant-like effect of acute systemic rapamycin administration, but this effect was observed in animal models of neurological pathologies as epilepsy [51,54], tuberous sclerosis complex [55], and Parkinson’s disease [56]. The behavioral discrepancies between our study and others might be due to the use of a genetic approach (mTOR-siRNA) compared with the pharmacological inhibition of mTOR (rapamycin), and/or the local (infralimbic cortex) versus the systemic administration, respectively. This marked behavioral effect following siRNAs unilateral infusion is in line with previous studies using this genetic approach [31].

We also failed to find any effect of the acute mTOR silencing in the IL cortex when preliminarily analyzing *state* anxiety using the open-field test. This finding contrasts with the anxiety-like behavior elicited by the chronic administration of mTOR inhibitors [52] and the overexpression of the mTOR inhibitor REDD1 in mPFC [41]. Besides, a single prenatal administration of rapamycin in mice induced increased anxiety in the adult offspring [57]. The appearance of anxiety in these studies compared to the lack of effect observed in our study could be due (1) to the systemic administration of mTOR inhibitors (compared to our local knockdown), (2) to the use of a wider battery of behavioral tests (including the elevated plus maze) [41,52], or (3) to the temporal appearance of this behavior, as some authors study the long-term behavioral effect [41,52,57] (compared to our acute studies). Overall, our data and those from previous reports reflect the complex and region-dependent effects of mTOR inhibition in the behavioral readouts, opening a new field of study in the treatment of depressive-related behaviors.

The importance of mTOR in the IL cortex in the neurobiology of depression could be complemented with the fact that the infusion of drugs as ketamine [38] and (2R,6R)-hydroxynorketamine [37] in this area in the rodent brain induce an antidepressant-like effect mediated by mTOR pathway activation. Moreover, this antidepressant-like effect is blocked by rapamycin infusion into the mPFC [14,37,58]. Ketamine administration in patients resistant to conventional antidepressant treatments induces a rapid improvement of depressive symptoms 2 h after administration that is maintained for up to 2 weeks [59,60,61]. However, preclinical studies indicate a lack of effect of rapamycin on the inhibition of the antidepressant-like effect of ketamine [62]. Moreover, a recent clinical study describes that the administration of rapamycin prolongs the antidepressant effect of ketamine [63], indicating that there is still a lot of work to be done to clarify the role of this signaling pathway in the mechanism of action of fast-acting antidepressant drugs.

The depressive-like behavior present after the acute mTOR-siRNAs infusion into the IL cortex correlates with the downregulation of BDNF expression in both ipsilateral IL and PrL cortices, as well as in the contralateral IL cortex. The BDNF downregulation observed in both the IL and PrL cortices may account for the neuronal network interaction of both areas [64,65]. Interestingly, a recent study reported a similar reduction of BDNF expression after the acute knockdown of the astrocytic glutamate transporter (GLAST/GLT-1) in the IL cortex of mice [31], associated also with a depressive-like phenotype. Several clinical and preclinical reports point to an impairment in neurotrophic factors, mainly BDNF, as the causal role of the atrophy observed in brain areas as PFC or hippocampus in patients diagnosed with major depression [66]. In this regard, BDNF levels are decreased in the post-mortem PFC samples from depressed patients [6,67,68], and in the PFC and hippocampus of rodent models of depression exposed to different types of stress [3,69].

The mPFC exerts top-down control over several limbic regions and brainstem nuclei, which, in turn, influence different aspects such as cognition and emotion, mental processes compromised in major depressive disorder. The outputs to the DRN are important to modulate the harmful effects of uncontrollable stress [70]. Therefore, the mPFC-DRN connection was analyzed in the present study, determining the extracellular levels of serotonin and glutamate in the DRN by in vivo microdialysis after acute mTOR-siRNAs infusion into the IL cortex, in parallel to the depressive-like behavior. Recent studies in depression-like mouse models reported an enhancement of the activity of the glutamatergic output from the IL cortex, leading to the inhibition of the serotonergic neurons in the DRN [31,71,72]. In contrast, our results did not show significant changes in serotonin or glutamate basal levels, in line with previous reports in other animal models of depression in relevant areas as the mPFC [49,73].

After the local infusion of the depolarizing agent veratridine, opposite changes were detected in the serotonin and glutamate levels in DRN with a significant decrease or increase, respectively, suggesting that the reduction of mTOR in the IL cortex may affect the readily releasable pool (RRP) of perisomatic serotonin neurons and glutamatergic terminals in the DRN. Rapamycin-mediated mTOR inhibition induces the depletion of synaptic vesicles from monoamine presynaptic terminals in the striatum in a macroautophagy-dependent manner [74]. In addition, a lower serotonin release promoted by veratridine in the infralimbic mTOR knockdown animals is similar to previous reports in animal models of depression as the olfactory bulbectomy [36], chronic corticosterone administration [49], and genetic models of depression as the conditional β-catenin knockout in GLAST-expressing cells [49]. This lower stimulation-induced neurotransmitter release [75] might be associated with an impaired ability to cope with stressful situations [76].

The increased glutamate release following veratridine observed in the animals after acute mTOR-siRNAs knockdown was also described after acute stress in the mPFC [77,78] or the amygdala [79]. Furthermore, the inactivation of mTORC1 in cultured glutamatergic hippocampal neurons is associated with an increased rate of spontaneous and asynchronous glutamate release [80]. In this sense, an enhancement of glutamatergic output activity from the IL cortex, causing inhibition of the serotonergic neurons mediated by GABAergic interneurons in the DRN, has been reported in mouse models of depression [31,71,72]. Moreover, the inactivation of the mPFC using muscimol is associated with the impairment of information processing, resulting in altered serotonergic activity and its behavioral consequences [70].

Regarding the effects of the local infusion of NBQX in DRN, the serotonin levels were not modified as previously described [81]. The reduction of glutamate extracellular levels following local AMPA inhibition does not have a straightforward explanation, since, to date, AMPA receptors have not been described in presynaptic localization in glutamatergic terminals. However, it has been reported an inhibitory role of AMPA receptors, leading to the presynaptic inhibition of GABAergic transmission in cerebellar cells, which may be mediated by G-protein-linked mechanisms [82].

Finally, some potential limitations could be considered. First, the use of only one behavioral test to preliminarily analyze the state anxiety may hinder a wider effect on anxiety of the acute infusion of mTOR-siRNA in the IL cortex. A second limitation is the lack of knowledge regarding the duration of the depressive-like effect of a single infusion of mTOR-siRNA into this cortical area. Finally, and associated with the latter, is the issue of whether the silencing of mTOR in the IL cortex for a longer period would have been able to induce other changes in the depressive-like behavior manifestations, such as the appearance of anhedonia. In this sense, further investigations will be needed to broaden the current data.

## 4. Materials and Methods

### 4.1. Animals

Male C57BL/6J mice (2–3 months old, 25–30 g, Charles River, Lyon, France) were housed under controlled conditions (22 ± 1  °C; 12 h light/dark cycle) with food and water available ad libitum, unless otherwise stated. All procedures involving the use of mice and their care followed the principles of the ARRIVE guidelines were carried out with the previous approval of the Animal Care Committee of the Universidad de Cantabria and according to the Spanish legislation and the European Communities Council Directive on “Protection of Animals Used in Experimental and Other Scientific Purposes” (86/609/EEC) (ref. No.: PI.05-17).

### 4.2. siRNAs

Two unmodified siRNAs against mTOR (mTOR-siRNAs) (GenBank accession #NM_020009.2) which specific sequences are: mTOR-siRNA1 sense: gaaggucacugaggauuuaTT, antisense: uaaauccucagugaccuucTT; and mTOR-siRNA2 sense: acccgggcgugaucaauaaTT, antisense: uuauugaucacgcccggguTT, were co-administered (Microsynth; Balgach, Switzerland). A non-sense siRNA (NS-siRNAs, sense: aguacugcuuacgauacggTT, antisense: ccguaucguaagcaguacuTT) with no homology to the mouse genome was used as a negative control (nLife Therapeutics, S.L. (La Coruña, Spain) as described (International patent application PCT/EP2011/056270)). In the intracerebral infusion, 40 μg (20 μg of each) of siRNAs were diluted in artificial cerebrospinal fluid (aCSF: 125 mM NaCl, 2.5 mM KCl, 1.18 mM MgCl_2_, 1.26 mM CaCl_2_, containing glucose 5%). aCSF was infused to control animals.

### 4.3. Drugs and Reagents

Veratridine and 2,3-dihydroxy-6-nitro-7-sulfamoyl-benzo(f)quinoxaline (NBQX) were purchased from Tocris Bioscience (UK). Veratridine (50 μM) [31,83] and NBQX (100 μM) [84,85], were infused through the intracerebral probe into the DRN of the animals.

[^33^P]α-dATP (2′ deoxyadenosine 5′-(α-thio) triphosphate at a specific activity of >2500 Ci/mmol was purchased from Perkin Elmer and GTPγS (10 μM) and used for the mTOR and BDNF in situ hybridization studies.

### 4.4. Acute Intracerebral siRNA Infusion

**Figure 5:**
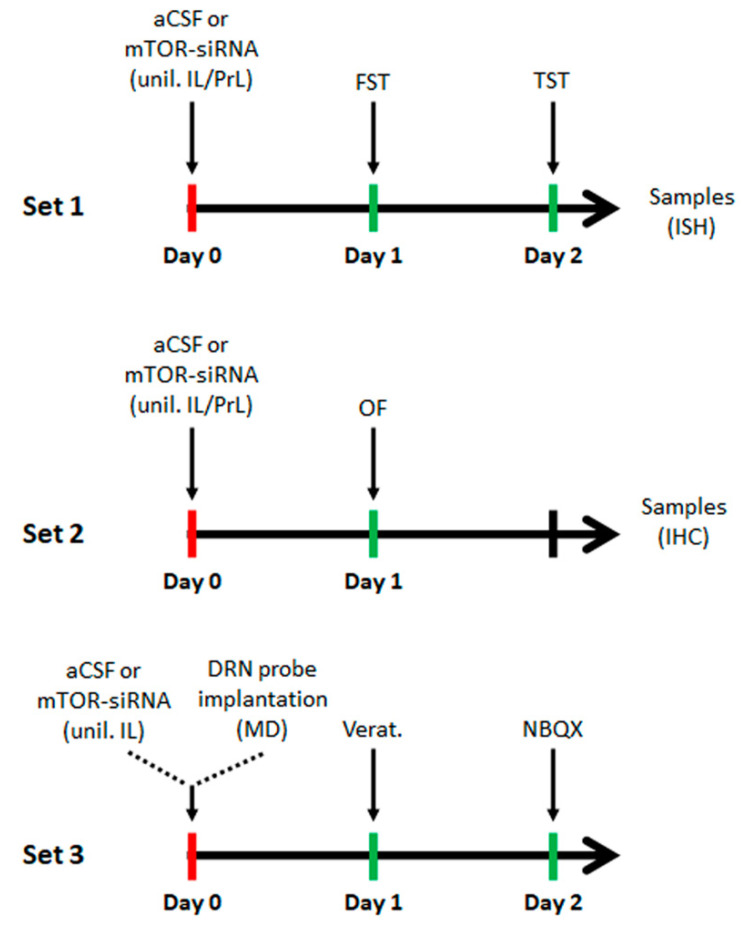
Experimental schedule of the different experimental procedures performed in this study. mTOR-siRNA or vehicle (aCSF) was unilaterally infused in the IL or PrL cortices for the behavioral experiments (sets 1 and 2), and in the IL cortex for the microdialysis studies (set 3). Samples were collected 48 h post-infusion (2 h after the last behavioral test for set 1). aCSF: artificial cerebrospinal fluid; siRNA: small interfering RNA; unil.: unilateral infusion; IL: infralimbic cortex; PrL: prelimbic cortex; DRN: dorsal raphe nucleus; FST: forced swimming test; TST: tail suspension test; OF: open-field test; ISH: in situ hybridization; IHC: immunohistochemistry; MD: microdialysis; Verat.: vetridine; NBQX: 2,3-dihydroxy-6-nitro-7-sulfamoyl-benzo(f)quinoxaline.

Mice were anesthetized with pentobarbital (40 mg/kg; i.p.) [31,49,86] and a single dose of the specific mTOR pool (40 μg/μL; 20 μg of each sequence) of siRNAs was stereotaxically and unilaterally infused [31], using a perfusion pump at 0.2 μL/min, in the infralimbic cortex (IL; coordinates in mm: anteroposterior-AP, +2.0; mediolateral-ML, –0.2 and dorsoventral-DV, –3.4), or the prelimbic cortex (PrL; AP, +2.0; ML, –0.2; DV, –2.0). The local infusion of this siRNA dose does not induce off-target effects [31,86,87]. A different set of animals was used for microdialysis studies. After siRNAs infusion, a microdialysis probe was implanted in the dorsal raphe nucleus (DRN) (Figure 5).

### 4.5. Behavioral Studies

All behavioral tests were performed between 10:00 a.m. and 3:00 p.m. by an experimenter blind to treatments. Mice were habituated for at least 1 h before testing. For the acute siRNAs treatment, animals were evaluated in two behavioral paradigms including the forced swimming test (FST) and the tail suspension test (TST), 24 and 48 h, respectively, after the single unilateral intracerebral infusion of mTOR-siRNAs or aCSF. In another animal cohort, the locomotor activity was assessed in the open-field test (OF) 24 h after the siRNAs or aCSF infusion (Figure 5).

#### 4.5.1. Forced Swimming Test (FST)

Mice were placed in cylinder tanks (30 × 20 cm) filled with water at 25 °C for a 6 min session, and the last 4 min were recorded. The time spent immobile was scored. At the end of the test, animals were immediately removed from the tank, dried off with a paper towel, and returned to their home cages [88].

#### 4.5.2. Tail Suspension Test (TST)

Mice were suspended 30 cm above the bench using adhesive tape placed approximately 1 cm from the tip of the tail. The total duration of immobility during a 6 min test was recorded using a video camera (Smart, Panlab) [87].

#### 4.5.3. Open-Field Test (OF)

Motor activity was measured in four Plexiglas open-field boxes 35 × 35 × 40 cm indirectly illuminated (25–40 luxes) to avoid reflection and shadows. The floor of the open field was covered with an interchangeable opaque plastic base that was replaced for each animal. Motor activity and the total time spent within the central area of the arena, to evaluate anxiety, were recorded for 15 min by a camera connected to a computer (Videotrack, View Point, Lyon, France) [87].

### 4.6. In situ Hybridization

Mice were killed by pentobarbital overdose and brains were rapidly removed, frozen on dry ice, and stored at −80 °C. Coronal tissue sections containing mPFC (14 μm thick) were cut using a microtome-cryostat (HM500-OM, Microm, Walldorf, Germany), thaw-mounted onto 3-aminopropyltriethoxysilane (Sigma-Aldrich, St. Louis, MO, USA)-coated slides, and kept at −80 °C until use. The oligoprobes used were: mTOR/4721-4766 (NM_020009) and BDNF/1188-1238 (NM_007540), respectively (Göttingen, Germany). Oligoprobes (2 pmol) were 3′-end labeled with [^33^P]-dATP (>2500  Ci/mmol; DuPont-NEN, Boston, MA, USA) using terminal deoxynucleotidyl transferase (TdT, Calbiochem, La Jolla, CA, USA), and the sections were incubated overnight with the labeled probes. Sections were then washed, air-dried, and exposed to films (Biomax MR, Kodak, Madrid, Spain) together with 14C microscales (Amersham, Buckinghamshire, UK) at 4 °C for 3 weeks. Films were analyzed and relative optical densities (ROD) were obtained using a computer-assisted image analyzer (MCID, Mering, Germany), as previously described [87,89]. The slide background and nonspecific densities were subtracted. ROD was evaluated in 2–3 duplicate adjacent sections from each mouse and averaged to obtain individual values along the anteroposterior axis.

### 4.7. mTOR Immunofluorescence

Animals were deeply anesthetized with sodium pentobarbital (40 mg/kg, i.p.) and transcardially perfused with 4% paraformaldehyde [90]. Brains were post-fixed for 4 h at 4 °C and cryoprotected with 30% sucrose in PBS. Free-floating coronal brain sections (40 µm thick) were processed for immunohistochemical experiments as follows. Sections were washed in PBS, blocked with a blocking solution (PBS containing 0.3% Triton-X-100 and 2% normal donkey serum) for 1 h at room temperature, and incubated with the primary antibody rabbit anti-mTOR (1:800, Cell Signaling Technology, Leiden, The Netherlands) in PBS and 2% normal donkey serum, overnight at 4 °C. After, sections were washed and incubated with the secondary donkey anti-rabbit Alexa Fluor 488 antibody (Invitrogen, Waltham, MA, USA) for 2 h at room temperature. After washing, sections were incubated with DAPI 1:1000 in PBS for 5 min and mounted using Vectashield. The fluorescent signal was detected using a Zeiss Axio Imager M1 fluorescence microscope, 12 bits B&W camera (AxioCam MRm). Cubes: GFP (Ex. 470/40–Em. 525/50). Objective: x40/NA 0.75. The relative immunoreactivity was measured as the mean densitometric measurement of the IL and PrL areas in silenced and control groups. The staining intensity was measured using the software ImageJ 1.52S (NIH, Bethesda, MD, USA). The image density was obtained by subtracting the density in the nonspecific condition (without primary antibody).

### 4.8. Intracerebral Microdialysis

Extracellular serotonin (5-HT) and glutamate (Glu) concentrations in DRN were measured by in vivo microdialysis, as previously described [31]. Briefly, one concentric dialysis probe (Cuprophan; 1.5-mm long) was implanted into the DRN (coordinates in mm: AP, −4.5; ML, 1.0; DV, −4.2, with a lateral angle of 20°) of pentobarbital-anesthetized mice. Microdialysis experiments were conducted 24 h (for intra-DRN infusion of veratridine; day 1) and 48 h (for intra-DRN infusion of NBQX; day 2) after surgery in freely moving mice by continuously perfusing probes with artificial cerebrospinal fluid (aCSF, containing 1 μM citalopram) at a rate of 1.64 μL/min. After a 180 min stabilization period, dialysate samples of 30 μL were collected every 20 min, six 20 min fractions were collected to obtain basal values (expressed as the concentration of neurotransmitter in the 30 μL sample), and another six samples after the local infusion of 50 μM veratridine (day 1) and 100 μM NBQX (day 2). 5-HT and Glu were determined by HPLC, as previously described [91]. The neurotransmitter levels (% vs. basal) were determined for every time point for each animal and the mean of the experimental groups was compared for 5-HT and Glu contents. The absolute basal levels of 5-HT (fmol/sample) and Glu (μmol/sample) were also compared among groups. After the experiments, mice were sacrificed, and brain tissue was processed according to standard procedures (cresyl violet staining) to verify the correct placement of the dialysis probe.

### 4.9. Statistical Analysis

Results are expressed as mean ± standard error of the mean (S.E.M.). The statistical analysis of the results was performed using Student’s *t*-test or two-way ANOVA, followed by the Bonferroni post hoc test. Graphs and statistical analyses were done using the GraphPad Prism software, version 6.1 (GraphPad Software Inc., San Diego, CA, USA). The level of significance was set at *p* < 0.05. The number of animals used in each experimental group is indicated in the Results section and Figure legends.

## 5. Conclusions

The present study demonstrates the causal link between mTOR expression in the infralimbic cortex and depressive-like state, with no clear implication in state anxiety-like behavior, at least in the open-field test. Here we demonstrate how an acute unilateral infusion of mTOR-siRNA into the infralimbic cortex, that induces a reduction of mTOR mRNA and protein expression in this area, is able to promote pro-depressive behavioral effects, accompanied with BDNF mRNA downregulation in the medial prefrontal cortex and an impairment of the serotonergic and glutamatergic neurotransmission in the ventromedial prefrontal cortex-dorsal raphe nucleus (vmPFC-DRN) pathway. These data support the importance of the mTOR pathway in the infralimbic cortex in the control of brainstem nuclei and the development of depressive-like behavioral despair. Our data are in accordance with previous findings on the importance of the mTOR pathway in major depression. Future experiments would be of interest to determine whether a sustained elimination of mTOR in the infralimbic cortex is able to promote a more pronounced behavioral phenotype (e.g., anhedonia).

## Data Availability

The data presented in this study are available on request from the corresponding author.

## Supplementary Materials

The following are available online at https://www.mdpi.com/article/10.3390/ijms22168671/s1.
