# Supplementary material for: mTOR Knockdown in the Infralimbic Cortex Evokes A Depressive-like State in Mouse
Source: Int J Mol Sci. 2021 Aug 12;22(16):8671. doi: 10.3390/ijms22168671 (PMC8395521; doi:10.3390/ijms22168671)
Supplement: Supplementary file 1 [file ijms-22-08671-s001.zip › ijms-1320093-SI.pdf]

International Journal of
*Molecular Sciences*

MDPI

*Article*

# mTOR knockdown in the infralimbic cortex evokes a depressive–like state in the mouse

**Emilio Garro-Martínez [1,2,3,$], M. Neus Fullana [1,4,5], Eva Florensa-Zanuy [1,2,3], Julia Senserrich [1,2,3], Verónica Paz [1,4,5], Esther Ruiz-Bronchal [1,4,5], Albert Adell [1,2], Elena Castro [1,2,3], Álvaro Díaz [1,2,3], Ángel Pazos [1,2,3], Analía Bortolozzi [1,4,5], Fuencisla Pilar-Cuéllar [1,2,3,*]**

**Supplementary Materials:**

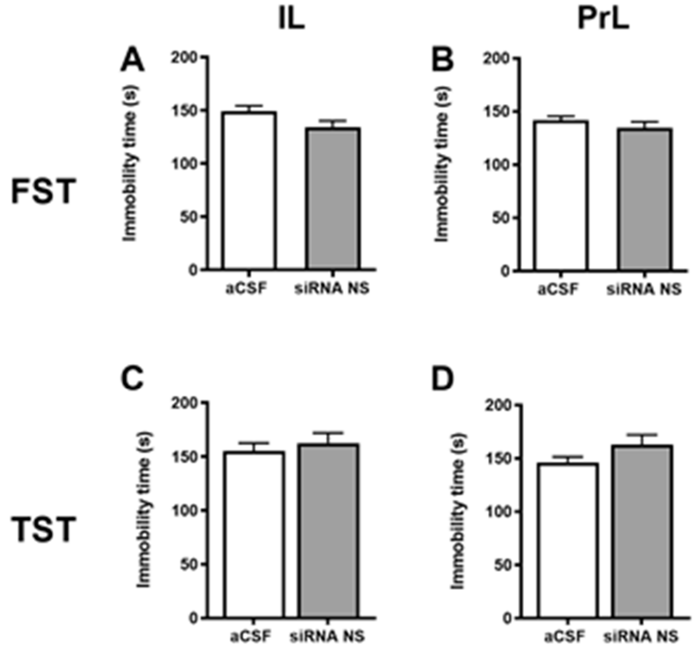


**Figure S1.** Effect of a single unilateral administration of non-sense siRNAs into the infralimbic and prelimbic cortices on depressive-like behavior. No changes were observed between animals infused in (A and C) the IL or (B and D) the PrL cortex in (A and B) the FST 24 h after acute unilateral infusion, or (C and D) the TST 48 h after infusion. Results are presented as mean±S.E.M. Student's t test. aCSF: artificial cerebrospinal fluid. n=4-6 animals per group.
